# Mitochondrial-Derived Peptides as Therapeutics and Biomarkers for Combating Vascular Aging and Associated Cardiovascular Diseases

**DOI:** 10.2174/011573403X375709250616134726

**Published:** 2025-06-20

**Authors:** Rooban Sivakumar, Arul Senghor Kadalangudi Aravaanan, Vinodhini Vellore Mohanakrishnan, Janardhanan Kumar

**Affiliations:** 1 Department of Biochemistry, SRM Medical College Hospital and Research Centre, SRM Institute of Science and Technology, SRM Nagar, Kattankulathur – 603203, Kanchipuram, Chennai, Tamil Nadu, India;; 2 Department of General Medicine, SRM Medical College Hospital and Research Centre, SRM Institute of Science and Technology, SRM Nagar, Kattankulathur – 603203, Kanchipuram, Chennai, Tamil Nadu, India

**Keywords:** Mitochondrial-derived peptides, vascular aging, oxidative stress, endothelial function, cardiovascular diseases, biomarkers

## Abstract

Vascular aging profoundly affects the onset of cardiovascular diseases in the elderly, mostly as a result of mitochondrial dysfunction. This review examines the protective roles of mitochondrial-derived peptides such as humanin, MOTS-c, and small humanin-like peptides in mitigating vascular aging. These peptides, encoded by mitochondrial DNA, are crucial for regulating apoptosis, inflammation, and oxidative stress, which have a major role in vascular health. MDPs have significant prospects as therapeutic and biomarker possibilities for the early diagnosis and intervention of vascular aging. MDPs influence the functions of endothelial and vascular smooth muscle cells by modulating critical signaling pathways, including AMPK, mTOR, and sirtuins. These pathways are essential for facilitating cellular metabolism, enhancing stress resilience, and prolonging longevity. Moreover, MDPs are essential in mitochondrial bioenergetics and dynamics, vital for mitigating endothelial dysfunction and enhancing vascular resilience. Furthermore, MDPs contribute to immunological modulation and the regulation of inflammatory responses, underscoring their potential therapeutic applications in the treatment of age-related vascular disorders. This review analyzes the various functions of MDPs in vascular health and their therapeutic importance, advocating for more studies to optimize their clinical benefits. By understanding the comprehensive roles and mechanisms of these multifunctional peptides, we can better appreciate their capacity to prevent and treat vascular aging and associated cardiovascular disorders. Future research should aim to further elucidate their therapeutic effects and optimize their clinical applications.

## 1 INTRODUCTION

With increasing age, arterial structure and function degenerate and eventually become stiff, arterial wall remodeling begins and vasodilatation decreases, all of which are features of vascular aging. Since this phenomenon predicts cardiovascular disease (CVD) and death, early identification of high-risk patients and suitable treatments is essential [1, 2]. Many diseases associated with age, such as atherosclerosis, hypertension, and heart failure, are strongly influenced by vascular aging, which is characterized by complex interactions between multiple structural and functional changes in the vascular system [3]. These changes increase elderly morbidity and cardiovascular risk [4]. Therefore, understanding the molecular pathways involved in vascular aging is key to counteracting the substantial associated health risks of age-related vascular disorders. As the world population ages, the incidence of cardiovascular events has increased; therefore, more knowledge regarding molecular alterations in blood vessels over time is necessary [5, 6].

Given their key roles in energy metabolism, oxidative stress management, and cellular homeostasis [7], mitochondria play crucial roles in the aging process. Mitochondrial malfunction is partly responsible for age-related declines in physiological activities and decreased energy due to its role as the main site of ATP generation [7, 8]. Vascular aging is intricately linked with mitochondrial dysfunction, and mitochondrial dysfunction itself contributes greatly to oxidative stress and inflammation in the aging vasculature and thus increases the risk of cardiovascular disease [9]. Many pathways, including mitochondrial DNA (mtDNA) leakage, oxidative stress, and impaired mitophagy, lead to this dysfunction [9-11].

Humanin, MOTS-c, and small humanin-like peptides (SHLP1-6) are mitochondrial-derived peptides (MDPs) that are bioactive microproteins encoded by short open reading frames in mitochondrial DNA [12, 13]. Recently, their importance in cellular processes such as apoptosis, inflammation, and oxidative stress, which are important for the maintenance of cellular health and functionality, has been reported [14]. MDPs are interesting in aging research because of their ability to increase mitochondrial activity, maintain metabolic homeostasis, protect against insulin resistance, and prevent the onset of age-related diseases [15], making them potential tools for the treatment of diseases such as diabetes and cardiovascular diseases. Vascular aging may be significantly influenced by MDPs through their effects on epigenetic systems that control vascular cell aging processes. Senescence and remodeling are increased during vascular aging, and MDPs might be associated with epigenetic modifications such as DNA methylation and histone changes, particularly H3K27 methylation, which is associated with vascular stiffness [16]. Importantly, it can also modulate gene expression by regulating important pathways, including the smooth muscle cell-mineralocorticoid receptor (SMC-MR) pathways, which are critical [16, 17].

Despite the understanding of MDPs in terms of their contributions to mitochondrial health, less is known about their potential specific roles in vascular aging. We understand very little about the role of MDPs in the regulation of endothelial function, inflammation, and mitochondrial dynamics in the aging vascular system. Herein, we review MDPs and their role in vascular oxidative stress and inflammatory signaling and highlight how they may alter endothelial function in the context of aging.

### 1.1 Literature Search

In this review, we have used a narrative synthesis method to analyze the available literature about mitochondrial-derived peptides in vascular aging and cardiovascular diseases. Literature search was conducted in 3 main databases, including PubMed, Scopus and Google Scholar. Animal, Human, and Cell line studies from January 2000 to October 2024 were included. Multiple search combinations were used including “mitochondrial-derived peptides”, “MDPs”, “Humanin”, “MOTS-c”, “vascular aging”, “vascular ageing”, “endothelial dysfunction”, “oxidative stress”, “cardiovascular diseases”, “atherosclerosis”, “inflammation” and “mitochondrial dysfunction”. The search operation included Boolean operator functions of AND and OR to enhance the search effectiveness. A detailed search string is given in Table **1**. The search implementation included limits for peer-reviewed articles and those available in English. We have included all original research articles, systematic reviews, meta-analyses and reviews that focus on oxidative stress, inflammation, mitochondrial dysfunction, or endothelial function in the context of aging. We have excluded editorials, opinions, and conference abstracts without full data.

## 2 MITOCHONDRIAL DYSFUNCTION AND VASCULAR AGING

Mitochondria are essential for vascular health due to their roles in energy production, oxidative stress regulation, and cellular homeostasis. However, mitochondrial dysfunction, a hallmark of aging, leads to impaired oxidative phosphorylation, excessive reactive oxygen species (ROS) generation, and accumulation of mitochondrial DNA mutations, contributing to endothelial dysfunction, vascular stiffness, and chronic inflammation [18-20]. These defects reduce nitric oxide (NO) bioavailability, impair endothelial function, and promote vascular aging by accelerating oxidative stress and inflammation [10, 18].

In endothelial cells, mitochondrial dysfunction disrupts NO production, weakening vascular tone and endothelial integrity, while increased ROS triggers oxidative stress and inflammatory cascades that exacerbate endothelial dysfunction [21, 22]. Similarly, in vascular smooth muscle cells (VSMCs), mitochondrial imbalances in fission and fusion impair contractility, increasing vascular stiffness and leading to hypertension and atherosclerosis [19, 20]. ROS-driven inflammatory responses in VSMCs further accelerate atherosclerotic plaque development and vascular aging [21, 22].

Energy deficits from impaired oxidative phosphorylation weaken EC and VSMC function, exacerbating vascular complications. Under diabetic conditions, hyperglycemia further depletes mitochondrial energy reserves, worsening microvascular dysfunction and aging [9, 23-26]. Dysfunctional mitochondria also initiate apoptosis *via* cytochrome c release and caspase activation, while excessive ROS causes necrosis, contributing to endothelial and smooth muscle cell loss, plaque instability, and progression of vascular diseases like atherosclerosis [27-30].

**Fig. (1):**
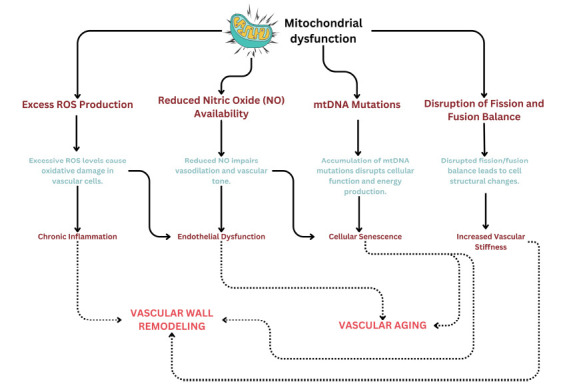
Impact of mitochondrial dysfunction on vascular aging.

Chronic systemic inflammation, driven by mitochondrial dysfunction and the senescence-associated secretory phenotype (SASP), disrupts extracellular matrix (ECM) remodeling and increases collagen deposition, promoting vascular stiffness and impairing blood flow [31-35]. With aging, defective mitophagy and reduced mitochondrial biogenesis lead to the accumulation of damaged mitochondria, exacerbating oxidative stress, inflammation, and vascular deterioration [36-40]. Given its profound impact on vascular function, targeting mitochondrial dysfunction remains a key therapeutic strategy for mitigating vascular aging (Fig. **1**).

## 3 MITOCHONDRIAL-DERIVED PEPTIDES AND ENDOTHELIAL FUNCTION

MDPs, such as Humanin, MOTS-c, and small humanin-like peptides (SHLPs), are synthesized within mitochondria and released into the bloodstream, where they function as endocrine-like signaling molecules. Their secretion and transport into circulation occur through several proposed mechanisms. One pathway involves non-classical secretion, where MDPs bypass the endoplasmic reticulum-Golgi system and are directly translocated across membranes [12]. Additionally, extracellular vesicles (EVs), including exosomes and microvesicles, have been identified as potential vehicles for MDP transport. These vesicles protect MDPs from enzymatic degradation and facilitate their targeted delivery to distant tissues. The ability of MDPs to circulate and influence systemic metabolic and vascular functions highlights their significance in inter-organ communication [9, 13].

Given their systemic presence, MDPs, particularly Humanin and MOTS-c, play essential roles in vascular health by modulating metabolic processes, maintaining endothelial function, and protecting against cellular stress. These peptides encoded by mitochondrial DNA modulate key cellular functions critical for preserving vascular integrity and function, including oxidative stress, apoptosis, and inflammation [41]. Furthermore, atherosclerosis, a major aspect of CAD, is protected by MOTS-c. Recent studies have revealed a correlation between decreased levels of MOTS-c and increased CAD severity and therefore, the possibility of utilizing MOTS-c in a prophylactic manner as a biomarker for detecting and treating CAD in early stages [42]. Additionally, decreased serum MOTS-c levels are associated with RHI in obese individuals, and decreased MOTS-c blood levels are positively correlated with decreased endothelial function, suggesting that MOTS-c is a potential therapeutic target for vascular complications occurring in obese individuals [43]. The function of MOTS-c for its use in diabetes is, moreover, advanced as it can also be used to repair myocardial damage in diabetic patients by attenuating apoptosis and angiogenesis and is thus a potential treatment for diabetic cardiomyopathy [44]. Like exercise, MOTS-c acts as an insulin sensitizer and can also activate AMPK as well as metabolic homeostasis and possibly prevent obesity-related vascularities [45, 46]. Similarly, humanin acts as a cytoprotective factor by increasing mitochondrial function and protecting cells from stress-induced damage, which is critical for maintaining vascular integrity [47]. Furthermore, humanin levels positively respond to physical exercise, which benefits vascular health, as shown by the modulation of humanin levels in athletes as a function of endurance exercise, indicating its role in energy metabolism and vascular performance [48]. Both humanin and MOTS-c offer protection against cellular stress, and studies have shown that they can help blunt damage in models of gentamicin-induced hair cell damage, suggesting a wider potential to protect cells and blood vessels from damage [44]. The levels of both peptides decrease with age and may play a role in vascular decline. Importantly, this finding implies that lifestyle interventions, such as exercise, could alter these peptides to overcome vascular aging and promote vascular health in advanced age [48].

### 3.1 Key Signaling Pathways for Endothelial Protection: AMPK, mTOR, and Sirtuins

**Fig. (2):**
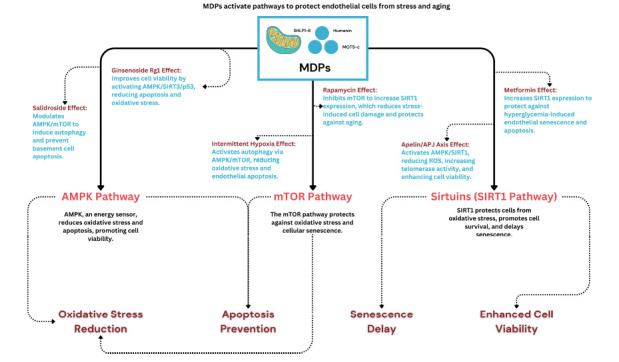
Impact of mitochondrial-derived peptides on vascular aging.

MDPs such as humanin and MOTS-c protect endothelial cells from oxidative stress, apoptosis, and senescence primarily through three central signaling pathways: the AMPK, mTOR, and sirtuin pathways. These effects include protective effects mediated through the AMPK pathway, a vital energy sensor of cells. For example, compounds such as ginsenoside Rg1 improve endothelial cell viability by activating the AMPK/SIRT3/p53 pathway to reduce both apoptosis and oxidative stress [49]. In addition, salidroside modulates the AMPK/mTOR pathway to induce autophagy, thereby reducing basement cell apoptosis induced by oxidative stress and enhancing the survival of cells [50]. Additionally, the Apelin/APJ axis maintains endothelial cell health through activation of the AMPK/SIRT1 pathway, resulting in decreased ROS level, increased telomerase activity, and improved cell viability, which delays somatic aging [51]. Additionally, the mTOR pathway is an important mediator that protects endothelial cells from sirtuin depletion, and the inhibition of mTOR reduces oxidative stress-induced senescence [52]. For example, the mTOR inhibitor rapamycin increases SIRT1 expression, protecting endothelial cells from aging and stress-related damage. Intermittent hypoxia has been shown, in tandem, to induce autophagy *via* the AMPK/mTOR pathway, which in turn protects against oxidative stress and endothelial apoptosis [53]. Sirtuins, specifically SIRT1, are critical for maintaining oxidative stress and cellular survival, and metformin has been found to increase SIRT1 expression to maintain hyperglycemia-induced endothelial senescence and apoptosis [54]. MDPs are transduced into endothelial cells through these pathways and thereby increase endothelial resilience to oxidative and metabolic stressors (Fig. **2**). Table **2** summarizes the key pathways and functions of mitochondrial-derived peptides, highlighting their impact on vascular health and aging.

### 3.2 Effects of MDPs on Mitochondrial Bioenergetics and Vascular Aging

MDPs significantly influence mitochondrial bioenergetics in endothelial cells, a central process in vascular aging. Humanin and MOTS-c are known MDPs that play protective roles, including maintaining mitochondrial function and cellular viability under stress conditions. Mitochondrial dysfunction, characterized by oxidative stress and decreased NO bioavailability, is typical for vascular aging [11, 21]. However, MDPs increase both mitochondrial efficiency and mitochondrial fatty acid β-oxidation, potentially with a delay in endothelial senescence [55]. However, MDPs play a multifaceted role in vascular aging. MDPs are commonly effective at decreasing oxidative stress and inflammation, but in some cases, they may also cause SASP by promoting the release of proinflammatory cytokines, especially IL-6 and TNF-α, which promote vascular senescence [56]. Importantly, this dual role emphasizes the importance of sustaining mitochondrial health by supporting vascular longevity, given that impaired mitophagy in endothelial cells has been shown to accompany aging-related cardiovascular diseases [57].

### 3.3 Distinct Effects of MDPs on Endothelial Cells and Vascular Smooth Muscle Cells

MDPs have opposing effects on endothelial cells and vascular smooth muscle cells, and they regulate the vascular response through differential roles in oxidative stress, apoptosis, and cellular signaling. MDPs such as MOTS‐c significantly increase mitochondrial respiration and ROS production in endothelial cells, increasing mitochondrial activity threefold and increasing ROS formation fivefold, which can promote cellular function but can also ultimately lead to ROS‐mediated endothelial dysfunction [58]. In contrast, in VSMCs, MDPs, such as mitofusin-2-related synthetic peptide (MRSP), primarily promote apoptosis *via* a mitochondrial-mediated pathway, resulting in caspase-3 activation and cytochrome c release and inhibition of the PI3K/Akt pathway. As a result, reduced VSMC proliferation may protect against vascular pathologies, such as neointimal hyperplasia [59]. Nevertheless, MDPs may be involved in cardiovascular health by affecting gene expression and transforming the cellular stimuli in a cell-type-specific manner to modulate oxidative stress, apoptosis, and inflammation, which are key elements that contribute to cardiovascular disease progression [13, 60]. The dual roles of these delivery systems are illustrated by their ability to promote oxidative stress in ECs and apoptosis in VSMCs, thus providing a rationale for targeted therapeutic approaches to optimize their therapeutic effects.

### 3.4 Interaction of MDPs with Age-related Pathways: mTOR and Sirtuins

MDPs also interact with known aging pathways, including mTOR inhibition and sirtuin activation, strongly affecting endothelial health. The mTOR pathway is a central regulator of cell growth, metabolism, and aging and is modulated by MDPs such as humanin and MOTS-c, which promote endothelial function when mTOR activity is decreased and support lifespan extension and cellular resilience [61]. In particular, humanin has protective effects against cellular stress and possibly modulates mTOR indirectly by promoting cellular stress response pathways [56]. In addition, MDPs increase the activity of sirtuins, particularly SIRT1, which are important for both cellular stress resistance and metabolic regulation. MDPs activate sirtuins, which promote mitochondrial health, decrease oxidative stress, and help strengthen endothelial resilience, which is important for vascular health [62]. In addition to improving endothelial function through sirtuin activation, endothelial function is further improved by decreasing insulin resistance and shifting the balance to an anti‐inflammatory state, protecting against endothelial dysfunction and promoting cardiovascular health [62]. MDPs show promise for improving endothelial health *via* these pathways, but their roles are complicated. At times, MDPs can exacerbate the SASP under certain conditions at large, which may in turn increase inflammation unless carefully regulated [56]. The dual nature of this molecule requires consideration of both the timing and dosage of therapeutic use. Furthermore, the involvement of MDPs in other pathways, including the AMPK and FOXO pathways, highlights that fully comprehending their mechanisms would facilitate the safe and effective use of MDPs for endothelial health [61].

## 4 MDPS IN VASCULAR INFLAMMATION AND IMMUNE MODULATION

MDPs, including Humanin, MOTS-c, and small humanin-like peptides (SHLPs), are recognized for their ability to affect inflammation and immune function during vascular aging. Encoded within mitochondrial DNA, these peptides are critical for countering `inflammaging', chronic low-grade inflammation, which is a central driver of vascular aging and related cardiovascular diseases. Mitochondrial dysfunction in aging blood vessels is exacerbated through chronic inflammation. With increasing age, mitochondria generate more reactive oxygen species, become functionally impaired, and release mitochondrial DNA from the organelle into the cytoplasm. This release activates inflammatory pathways such as the cGAS-STING pathway [10, 63], looping back to a cycle of inflammation that further damages the mitochondria and even the vascular cells themselves. A primary contributor to endothelial dysfunction, arterial stiffness, and atherosclerosis, increasing the risk for CVDs, is this ongoing cycle [64].

The ability of MDPs to modulate pro- and anti-inflammatory cytokine production in vascular tissues has been shown. For example, humanin and MOTS-c block the NLRP3 inflammasome, key inflammatory mediators, from secreting IL-1β and IL-18 and thus reduce inflammation. Moreover, these peptides simultaneously increase the expression of anti-inflammatory cytokines to achieve homeostasis and, subsequently, reduce the inflammatory burden on the vascular system [13, 60]. As MDPs are involved in the regulation of cytokines, they function as antioxidants of the type that reduce low-grade, chronic inflammation often found in the aging vasculature. MDPs enhance mitochondrial function and reduce oxidative stress, suggesting promising strategies for mitigating inflammation-related aging [65] and, consequently, for preserving the function of the vascular system the elderly individuals [66].

Both humanin and MOTS-c have been shown to have anti-inflammatory effects on endothelial cells and vascular smooth muscle cells. These peptides have been shown to decrease oxidative stress and inhibit inflammatory cytokine production, resulting in the protection of vascular cells from stress-induced damage and decreasing markers of inflammation [67, 68]. During aging, MDPs modulate the expression of adhesion molecules and cytokines to regulate immune cell infiltration and activation within the vasculature through the control of the recruitment and activation of immune cells. This regulation is essential for limiting excessive inflammatory responses and the age-related rise in vascular inflammation [69, 70]. MDPs have therapeutic potential in vascular inflammatory disease; however, strategies that increase MDP levels or replicate their effects still need to be developed. It encompasses MDP analog development, mitochondrial transplantation, and targeted delivery to inflamed vascular tissues. These strategies seek to leverage the cytoprotective and anti-inflammatory functions of MDPs for the management and treatment of CVDs and provide new entry points for interventions in vascular aging [13, 60].

## 5 MDPS AS BIOMARKERS OF VASCULAR AGING

Owing to their critical involvement in the regulation of apoptosis, inflammation, and oxidative stress, two circulating MDPs (humanin and MOTS‐c) have the potential as biomarkers of vascular aging and endothelial dysfunction. Because MDPs are encoded by mitochondrial DNA and their levels act as a marker of the functional state of mitochondria as well as a systemic marker of vascular health, they are ideal early and serial markers for mitochondrial dysfunction, a common consequence of aging and vascular endothelial damage [31, 71]. Humanin and MOTS-c, in particular, appear to be promising biomarkers of vascular aging. Lower levels of MOTS-c have been linked to cardiovascular diseases such as coronary artery disease, where a lower MOTS-c level is associated with more severe disease and impaired endothelial function, especially in obese children [42, 43]. Given its protective role in cardiovascular health and its roles in cell survival and stress response, humanin is a valuable marker for monitoring the effects of lifestyle interventions on vascular aging [48].

The levels of both peptides decrease with age in parallel with vascular aging and associated disease. This decline is associated with increased cellular senescence, chronic inflammation, and oxidative stress, which are themselves important contributors to vascular aging and decline. The cardioprotective properties of MDPs that counteract these harmful processes make them potential biomarkers that may signal early disease progression in aging populations [70, 72]. Additionally, MDPs are being investigated as both biomarkers and new therapeutic targets for the manipulation and control of cardiovascular diseases because of their importance in maintaining vascular integrity and function. Modulation of MDP levels has the potential to provide new therapeutic avenues to control cardiovascular diseases [60]. The use of MDPs in clinical practice is, however, hampered by variability in sample collection and processing, which calls for the development of standardized protocols and reliable assays to obtain consistent results. MDP-based therapies and diagnostics must also navigate the regulatory and ethical hurdles associated with introducing a new class of drugs and analytics. However, future progress in mitochondrial medicine is opening the door for MDP measurements to be included in routine diagnostics and treatment options, while creating opportunities to integrate these measurements clinically [73, 74]. Tables **3** and **4** show the list of recent studies, summarizing key findings from articles on Mitochondria-derived peptides in the context of vascular aging and related conditions [76-80].

## 6 THERAPEUTIC POTENTIAL OF MDPS

Humanin, MOTSc, and SHLP1–6 have all shown great potential as therapeutics for the treatment of age-related vascular diseases. The peptides encoded inside mitochondrial DNA and their crucial roles in maintaining mitochondrial function and providing protection against stress conditions such as oxidative stress, inflammation, and apoptosis that occur during vascular aging have been identified [60]. MOTS-c has been shown to reduce vascular calcification and myocardial remodeling *via* the AMPK signaling pathway, which is critical for fighting against vascular aging [75]. Similarly, decreased MOTS-c levels in coronaries are also correlated with increased severity of CAD, suggesting their role as biomarkers and potential therapeutic targets [42]. In addition, humanin has cytoprotective effects in response to oxidative stress-induced apoptosis in endothelial cells and is found at relatively high concentrations in unstable atherosclerotic plaques, suggesting a protective role against inflammation and apoptosis at these sites [80, 81].

The therapeutic scope of MDP therapies is further amplified by combining MDP therapies with other anti-aging interventions, such as senolytics and caloric restriction mimetics. The bound pesticide has the potential to enhance mitochondrial function, improve ATP production, decrease oxidative stress, and prolong the cellular lifespan [82]. Caloric restriction mimetics induce autophagy and mitochondrial biogenesis, mimicking the effects of caloric restriction without reducing caloric consumption, and senolytics can eliminate senescent cells, lowering ongoing inflammation and resulting in improved tissue function [83]. In addition, mitochondrion-targeted antioxidants, such as MitoQ, and long-term lifestyle changes, including lifelong aerobic exercise, also benefit from improved endothelial function and reduced mitochondrial oxidative stress [84, 85]. Mitochondrial pathways, such as the involvement of Sirt3, are emerging as potential targets to combat vascular aging [75, 86]. However, integrating these therapies has several advantages, but the complexity of interactions between various pathways and the necessity of personalized approaches depending on individual mitochondrial characteristics still constitute difficulties.

While the therapeutic scope of MDPs is promising, several challenges limit their clinical applicability. MDPs are peptides prone to rapid enzymatic degradation and have poor intrinsic stability in the bloodstream, making bioavailability the main problem. Advanced drug delivery systems such as liposomal encapsulation, biodegradable nanoparticles, and peptide structure modifications are under investigation to enhance their stability and systemic availability [41]. Though preliminary studies suggest MDPs have a favorable safety profile, extensive toxicological evaluations are necessary to assess potential immunogenicity and off-target effects, especially with long-term use or at higher doses. Continued research is needed to optimize these combinations, as they need to be addressed to ensure that they are safe and effective for different populations [41].

## 7 COMMENTS ON FUTURE DIRECTIONS AND LIMITATIONS

Future studies of mitochondrial-derived peptides will need to translate these findings to the development of targeted therapies that improve their bioavailability, stability, and specificity through the use of peptide analogs or synthetic derivatives. To establish a causal link, longitudinal studies that evaluate MDP levels and vascular health markers, such as endothelial function and vascular stiffening, are needed. Investigations of this type could establish the likelihood of MDPs acting as early biomarkers of vascular aging and refine predictive applications. Moreover, mitochondrion-targeted drug delivery systems, such as nanoparticle or exosome-based technologies, can be used to increase the precision and efficiency of MDP therapy *via* direct delivery to damaged vascular tissues. However, MDPs promise a way out of vascular aging, but there are significant limitations. MDPs have been promising *in vitro* and animal studies, but the precise mechanistic pathways involved remain incompletely understood in humans. However, this gap calls for more clinical and translational studies of these mechanisms and ultimately to determine whether MDPs have therapeutic relevance in vascular health. Furthermore, the heterogeneity in individual responses to MDPs, driven by genetic, lifestyle, and environmental factors, combined with a lack of racial representation in MDP clinical trial studies, highlights the generalizability of therapeutic applications, underscoring the necessity for personalized strategies and the eventual execution of large-scale trials on a range of individuals from different populations. Finally, although MDP biomarker testing methods can be standardized, practical limitations such as variability in assay methods and sample handling and their reproducibility and clinical utility limit this possibility. MDPs such as Humanin can attenuate the senescence-associated secretory phenotype (SASP), yet their dual effects pose challenges. While beneficial at moderate doses, high doses or prolonged exposures can paradoxically activate NF-kB signaling pathways. This activation may lead to an increased secretion of pro-inflammatory cytokines, exacerbating inflammation rather than reducing it. Such effects are particularly detrimental in chronic conditions like atherosclerosis and other age-related vascular disorders, where chronic inflammation is a major concern. These results show how dose optimization and therapeutic timing can be critical to MDP application. Combining MDPs with other anti-aging interventions is somewhat complicated and requires a thorough understanding of how to obtain dosages correctly, sequence them, and maximize synergistic effects while minimizing adverse interactions. To fully capitalize on the potential of MDPs for the management of vascular aging and improved cardiovascular health in the aging population, further research that addresses these challenges is needed.

## CONCLUSION

Although mitochondrial-derived peptides are part of the future, they are promising modulators of vascular aging as they help with mitochondrial functions, reduce oxidative stress, modulate inflammation, and maintain cellular homeostasis. With increasing evidence that mitochondrial dysfunction plays an important role in vascular aging and associated pathologies, MDPs represent a novel way of preventing these processes, which could postpone the onset and progression of age-related cardiovascular disease. The therapeutic potential of MDPs is underscored by their dual effects on endothelial and vascular smooth muscle cells, although the effects are complex, including protective and proinflammatory actions in specific conditions. The complexity of these materials indicates that to maximize their efficacy and safety in clinical applications, they require optimized dosing, delivery mechanisms, and combination strategies. Although promising as biomarkers and therapeutic agents, the roles of MDPs require further study, and methods for standardizing biomarker assays and providing protocols that yield consistent therapeutic results need to be established. Future studies of vascular health MDPs will almost certainly unveil unique insights and approaches toward managing cardiovascular health in aging populations, and facilitate the incorporation of these observations into clinical practice.

## Figures and Tables

**Table 1:** Detailed literature search strings.

**Database**	**Search Strategy**	**Additional Steps**
PubMed	(“Mitochondrial-derived peptides”[All Fields] OR “Humanin”[All Fields] OR “MOTS-c”[All Fields] OR “Small Humanin-like Peptides”[All Fields]) AND (“Vascular Aging”[All Fields] OR “Endothelium, Vascular”[MeSH] OR “Oxidative Stress”[MeSH] OR “Inflammation”[MeSH] OR “Cardiovascular Diseases”[MeSH] OR “Mitochondria”[MeSH])	Manual Title and Abstract screening was conducted to retrieve relevant articles.
Scopus	TITLE-ABS-KEY (“mitochondrial-derived peptides” OR “Humanin” OR “MOTS-c” OR “small humanin-like peptides”) AND (“vascular aging” OR “endothelial dysfunction” OR “oxidative stress” OR “cardiovascular diseases” OR “atherosclerosis” OR “mitochondrial dysfunction”)	
Google Scholar	“mitochondrial-derived peptides” OR “Humanin” OR “MOTS-c” OR “small humanin-like peptides” AND “vascular aging” OR “endothelial dysfunction” OR “oxidative stress” OR “cardiovascular diseases” OR “atherosclerosis” OR “mitochondrial dysfunction”	

**Table 2:** Summary of key pathways and functions of mitochondrial-derived peptides (MDPs) in vascular aging.

**Peptide**	**Pathway**	**Function in Vascular Health**	**Impact on Vascular Aging**
Humanin	NRF2, NF-κB	Antioxidant, Anti-inflammatory	Reduces oxidative stress and inflammation, mitigates endothelial dysfunction
MOTS-c	AMPK, mTOR	Anti-apoptotic, Insulin Sensitizing	Enhances cellular metabolism, prevents vascular stiffness
SHLPs	AMPK, NRF2	Antioxidant, Anti-inflammatory	Decreases vascular inflammation, enhances cellular resilience

**Table 3:** Overview of animal studies on MDP levels and their role in vascular aging and related conditions.

**Study**	**Year**	**Study** **Design**	**Condition** **(Vascular Aging Context)**	**MDP Levels**	**Key Findings**	**Limitations**	**Future** **Directions**
Cobb *et al*. [62]	2016	Experimental (Mice, *in vitro*)	Aging-related cellular models	Decreased SHLP2 with age	SHLP2 levels decrease with age; SHLP2 enhances cell viability, reduces apoptosis, and improves mitochondrial metabolism.	Limited to animal and cell models	Investigate SHLP2 as a potential target for anti-aging therapies, especially in mitochondrial health.
Kim *et al*. [75]	2018	*In vitro*	Cellular senescence model	Elevated HN, MOTS-c	Senescent cells showed higher mitochondrial numbers and increased HN and MOTS-c, supporting cellular energetics.	*In vitro* findings may not fully translate to *in vivo*	Investigate MDP roles in senescent cells across vascular aging *in vivo*, focusing on SASP modulation
Wei *et al*. [76]	2020	Experimental (Rat)	Vascular calcification model	Increased MOTS-c	MOTS-c treatment attenuated vascular calcification *via* AMPK activation and decreased AT-1 and ET-B receptor expression.	Limited to the animal model	Explore MOTS-c effects in human vascular diseases

**Table 4:** Overview of human studies on MDP levels and their role in vascular aging and related conditions.

**Study**	**Year**	**Study Design**	**Condition** **(Vascular Aging Context)**	**MDP Levels**	**Key Findings**	**Limitations**	**Future** **Directions**
Ramanjaneya *et al*. [77]	2019	Cross-sectional	Type 2 Diabetes (T2D), prediabetes	Decreased HN, MOTS-c	Both HN and MOTS-c levels were significantly lower in T2D and correlated with HbA1c and glucose levels.	Cross-sectional design limits causality	Examine interventions to restore MDP levels in T2D and related vascular aging outcomes.
Gidlund *et al*. [78]	2016	Clinical Intervention	Impaired Glucose Regulation (IGR)	Increased HN in the muscle	12-week resistance training raised HN levels in muscle, potentially improving glucose metabolism.	Small sample size and limited to resistance training	Study the effects of other types of exercise on MDPs and glucose regulation in vascular aging.
Bolignano *et al*. [79]	2024	Prospective Cohort	Chronic Hemodialysis (ESKD) patients	U-shaped Humanin	Humanin levels displayed a U-shaped association with CV outcomes; both low and high levels were associated with higher CV risk.	Small sample and pilot study	Further investigate Humanin’s predictive value in CV risk and mortality in larger, diverse cohorts.

## LIST OF ABBREVIATIONS

CVD: Cardiovascular Disease
ECM: Extracellular Matrix
EVs: Extracellular Vesicles
IGR: Impaired Glucose Regulation
MDPs: Mitochondrial-derived Peptides
mtDNA: Mitochondrial DNA
NO: Nitric Oxide
ROS: Reactive Oxygen Species
SASP: Senescence-associated Secretory Phenotype
SHLPs: Small Humanin-like Peptides
T2D: Type 2 Diabetes
VSMCs: Vascular Smooth Muscle Cells

## DECLARATIONS

The authors are non-native English speakers. So they have used Quillbot to enhance the language of this work and subsequently reviewed and edited the content, taking full responsibility for the final publication.
